# Modeling Bacterial Attachment Mechanisms on Superhydrophobic and Superhydrophilic Substrates

**DOI:** 10.3390/ph14100977

**Published:** 2021-09-26

**Authors:** T. Brian Cavitt, Niyati Pathak

**Affiliations:** Department of Chemistry and Biochemistry, Lipscomb University, One University Park Drive, Nashville, TN 37204, USA; npathak@mail.lipscomb.edu

**Keywords:** bacterial-substrate interaction, Gibbs interaction energy, surface energy, extended DLVO theory, superhydrophobic, superhydrophilic

## Abstract

Superhydrophilic and superhydrophobic substrates are widely known to inhibit the attachment of a variety of motile and/or nonmotile bacteria. However, the thermodynamics of attachment are complex. Surface energy measurements alone do not address the complexities of colloidal (i.e., bacterial) dispersions but do affirm that polar (acid-base) interactions ($\Delta G^{AB}$) are often more significant than nonpolar (Lifshitz-van der Waals) interactions ($\Delta G^{LW}$). Classical DLVO theory alone also fails to address all colloidal interactions present in bacterial dispersions such as $\Delta G^{AB}$ and Born repulsion ($\Delta G^{Born}$) yet accounts for the significant electrostatic double layer repulsion ($\Delta G^{EL}$). We purpose to model both motile (e.g., *P. aeruginosa* and *E. coli*) and nonmotile (e.g., *S. aureus* and *S. epidermidis*) bacterial attachment to both superhydrophilic and superhydrophobic substrates via surface energies and extended DLVO theory corrected for bacterial geometries. We used extended DLVO theory and surface energy analyses to characterize the following Gibbs interaction energies for the bacteria with superhydrophobic and superhydrophilic substrates: $\Delta G^{LW}$, $\Delta G^{AB}$, $\Delta G^{EL}$, and $\Delta G^{Born}$. The combination of the aforementioned interactions yields the total Gibbs interaction energy ($\Delta G^{tot}$) of each bacterium with each substrate. Analysis of the interaction energies with respect to the distance of approach yielded an equilibrium distance ($d_{eq}$) that seems to be independent of both bacterial species and substrate. Utilizing both $d_{eq}$ and Gibbs interaction energies, substrates could be designed to inhibit bacterial attachment.

## 1. Introduction

Reducing nosocomial infection rates is a significant concern in the medical community. Many of the microorganisms contributing to the aforementioned infections originate in biofilms. Biofilm mitigation has been studied in depth including the disruption of the initial primary colonization [1,2,3,4,5,6,7,8,9,10,11,12,13,14,15,16,17,18].

To disrupt primary colonization, bacteria may be treated as a colloidal dispersion which abides by Derjaguin, Landau, Verwey, and Overbeek (DLVO) theory which relates dispersion stability, $\Delta G^{tot}$, to Gibbs interaction energy shown in Equation (1) [19,20,21,22,23,24]:

(1) $$\Delta G^{tot}=\Delta G^{LW}+\Delta G^{EL}$$

where $\Delta G^{LW}$ describes the Lifshitz-van der Waals (i.e., nonpolar) adhesive interactions between the bacterium and substrate and $\Delta G^{EL}$ describes the electrostatic double layer repulsive force of the bacterium with the substrate. Classical DLVO theory can account for dilute bacterial dispersions in a solvent of known zeta potential (ζ) and ionic strength (*I*) and requires a smooth, homogeneous substrate surface for the substrate interactions [23,25,26,27]. Superhydrophobic and superhydrophilic surfaces can provide a smooth, homogeneous surface energy compliant with classical DLVO theory [28,29]. However, classical DLVO theory does not account for either acid-base adhesive interactions ($\Delta G^{AB}$) or Born repulsion ($\Delta G^{Born}$) between the bacterium and the substrate; extended DLVO theory details the contribution of both $\Delta G^{AB}$ and, in some cases, $\Delta G^{Born}$ as shown in Equation (2) [17,19,30,31,32]:

(2) $$\Delta G^{tot}=\Delta G^{LW}+\Delta G^{AB}+\Delta G^{EL}+\Delta G^{Born}$$

In both classical DLVO and extended DLVO theories, the Gibbs interaction energies are defined in terms of the bacterial geometry and the distance between the bacterium and substrate [17,22,24,33]. Understanding the bacterium-substrate interaction is critical for designing substrates that are inherently biofilm resistant via inhibition of primary colonization.

Therefore, the purpose of this paper is to model the bacterium-substrate (superhydrophobic and superhydrophilic) Gibbs interaction energies with respect to bacterial geometry to determine an equilibrium distance ($d_{eq}$) between a bacterium and substrate. To assess motility, we modeled the behavior of two nonmotile bacteria [*S. epidermidis* (SE) and *S. aureus* (SA)] and two motile bacteria [*E. coli* (EC) and *P. aeruginosa* (PA)]. Using established protocols based on extended DLVO theory, we calculated each bacterium’s component Gibbs interaction energies ($\Delta G^{LW}$, $\Delta G^{AB}$, $\Delta G^{EL}$, and $\Delta G^{Born}$) and the total Gibbs interaction energy ($\Delta G^{tot}$) with respect to bacterial geometry. Having thereby established the fractional contribution of the component interaction energies with respect to the total interaction energy and assuming constancy for moderate bacteria-substrate distances, we then graphically determined $\Delta G^{tot}$ and $d_{eq}$ of bacteria-substrate aqueous interactions. By accounting for each bacterium’s surface area, we were able to determine the percent contribution of each extended DLVO component interaction energy which can then be used to better tailor biofilm resistant substrates.

## 2. Results

### 2.1. Surface Energy Determination of Gibbs Interaction Energies

After aggregating surface energy data necessary for the numerical experiments, we calculated nonpolar (i.e., Lifshitz-van der Waals) adhesive interactions ($\Delta G^{LW}$) and polar (i.e., acid-base) adhesive interactions ($\Delta G^{AB}$) for each aqueous bacterium [*P. aeruginosa* (PA), *S. aureus* (SA), *S. epidermidis* (SE), and *E. coli* (EC)] with both superhydrophobic and superhydrophilic substrates. The total Gibbs interaction energy ($\Delta G^{tot}$) was calculated for each bacterium with each substrate. The results of the numerical experiments are shown below in Table 1 and Table 2 for the superhydrophobic and superhydrophilic substrates, respectively.

**Figure 1:**
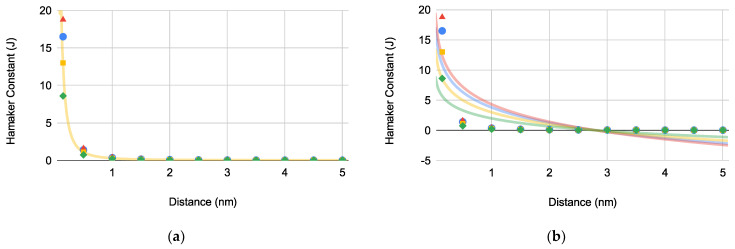
Hamaker constants ($\times 10^{-21}$) versus distance for PA (blue circles), EC (green diamonds), SA (red triangles), and SE (orange squares): (**a**) bacterial Hamaker constant determination ($H_{1}^{PA}=3.46\times 10^{-22}\;J$, $H_{1}^{SA}=3.94\times 10^{-22}\;J$, $H_{1}^{SE}=2.73\times 10^{-22}\;J$, and $H_{1}^{EC}=1.80\times 10^{-22}\;J$) and (**b**) bacterial equilibrium distance ($d_{eq}$) determination where $d_{eq}$ is 2.80 ± 0.00479 nm (*N* = 4).

The Hamaker constants ($H_{i}$ where *i* describes the interacting materials) for each bacterium was determined using Equation (3) with respect to the bacterium-substrate approach distance (*d*) to obtain the graphically determined $H_{1}$ (Figure 1a) and equilibrium distance ($d_{eq}$) for each bacterium (Figure 1b):

(3) $$H_{1}=\frac{\gamma_{1}^{LW}}{32\pi d^{2}}$$

where $\gamma_{1}^{LW}$ is the nonpolar surface energy component of the bacterium [33].

The graphically determined $H_{1}$ for EC correlates with literature value indicating that our calculated values in Figure 1a are representative of their respective bacteria [14]. Based on Figure 1b, $d_{eq}$ for each bacterium was determined to be 2.80 ± 0.00479 nm (*N* = 4).

**Figure 2:**
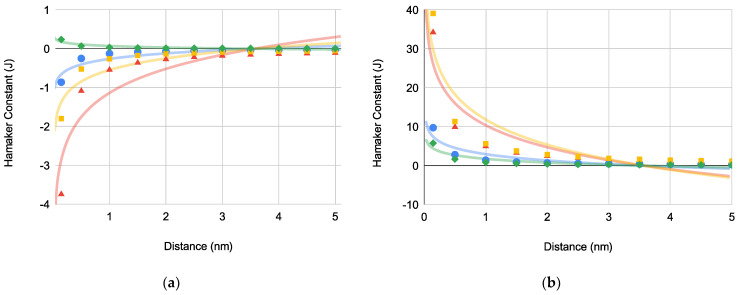
Hamaker constants ($\times 10^{11}$) versus distance to determine equilibrium distances accounting for bacteria-substrate interaction geometries and bacterial surface area for PA (blue circles), EC (green diamonds), SA (red triangles), and SE (orange squares) with: (**a**) superhydrophobic substrates where $d_{eq}$ is 3.58 ± 0.00479 nm (*N* = 4) and (**b**) superhydrophilic substrates where $d_{eq}$ is 3.58 ± 0.00250 nm (*N* = 4).

Furthermore, we determined $H_{i}$ for the bacterial $\Delta G^{LW}$ interaction with both superhydrophobic (Figure 2a) and superhydrophilic (Figure 2b) substrates using Equation (4) which accounts for the spherical-plane and spherocylindrical-plane interaction geometries and bacterial surface area:

(4) $$H_{i}=\frac{-\frac{\Delta G_{i}^{LW}r}{6d^{2}}}{A_{1}}$$

where $\Delta G_{i}^{LW}$ is the nonpolar bacterium-substrate interaction energy, r is the bacterium radius, d is the approach distance, and $A_{1}$ is the surface area of the bacterium. Interestingly, $d_{eq}$ for each bacterium increased to 3.58 ± 0.00267 nm (*N* = 8).

Considering the bacteria-water-superhydrophobic substrate interaction (indicated as a subscripted 132) in Table 1, $\Delta G_{132}^{LW}$ were slightly positive with the exception of EC which was slightly negative. All of the $\Delta G_{132}^{LW}$ were relatively small in magnitude relative to $\Delta G_{132}^{AB}$. Interestingly, upon comparing $\Delta G_{132}^{tot,sum}$ [Equation (5)] with $\Delta G_{132}^{tot,calc}$ [Equation (6)], no differentiation is observed for superhydrophobic substrates.

(5) $$\Delta G_{132}^{tot,sum}=\Delta G_{132}^{LW}+\Delta G_{132}^{AB}$$

(6) $$\Delta G_{132}^{tot,calc}=\gamma_{12}^{tot}-\gamma_{13}^{tot}-\gamma_{23}^{tot}$$

Given the assumption of the associated water interface with the substrate [34,35,36], the aqueous bacteria-superhydrophilic substrate interactions (indicated as a subscripted 13) are also remarkable with $\Delta G_{13}^{LW}$, $\Delta G_{13}^{AB}$, $\Delta G_{13}^{tot,sum}$, and $\Delta G_{13}^{tot,calc}$ all presenting as favorable/spontaneous with $\Delta G_{13}^{AB}$ having the largest magnitude. Unlike the bacteria-water-superhydrophobic substrate interactions, $\Delta G_{13}^{tot,sum}$ [Equation (7)] and $\Delta G_{13}^{tot,calc}$ [Equation (8)] are differentiated for each bacterial species with EC having an inverse relationship (i.e., $\Delta G_{13}^{tot,sum}$ > $\Delta G_{13}^{tot,calc}$) relative to the other bacteria.

(7) $$\Delta G_{13}^{tot,sum}=\Delta G_{13}^{LW}+\Delta G_{13}^{AB}$$

(8) $$\Delta G_{13}^{tot,calc}=\gamma_{13}^{tot}-\gamma_{1}^{tot}-\gamma_{3}^{tot}$$

The plot of the bacteria-substrate Hamaker constant versus distance for both superhydrophobic ($H_{132}$) and superhydrophilic ($H_{13}$) substrates (Figure 2a,b, respectively) yielded what appears to be identical equilibrium constants ($d_{eq}$) for both substrates.

### 2.2. Extended DLVO Determination of Gibbs Interaction Energies

The four individual interaction components of the extended DLVO theory (e.g., $\Delta G^{LW}$, $\Delta G^{AB}$, $\Delta G^{EL}$, and $\Delta G^{Born}$) need to be evaluated with respect to the distance separating bacterium (e.g., PA, SA, SE, and EC) from either superhydrophobic or superhydrophilic substrates after which $\Delta G^{tot}$ may be calculated. As before, x-intercepts will indicate the $d_{eq}$ of bacterium to substrate. Each dataset is given a logarithmic trendline for consistency with previous experiments and to obtain the graphical $d_{eq}$. Bacterial geometries are accounted for in terms of sphere-plate interactions where applicable.

**Figure 3:**
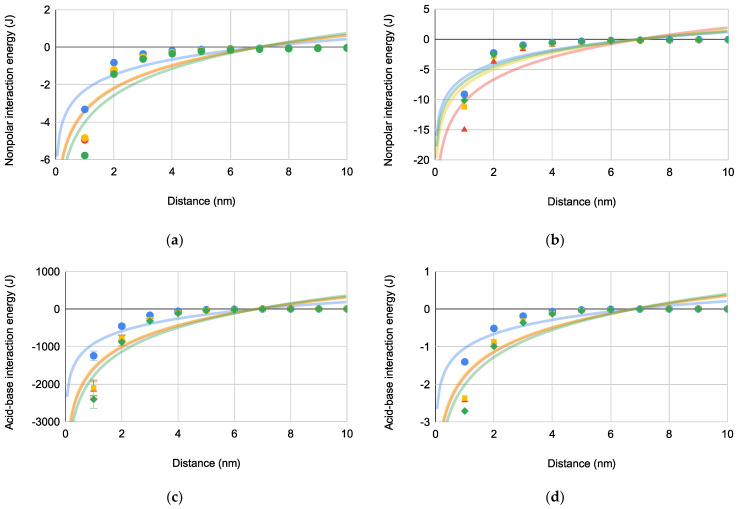
Bacteria-substrate Gibbs interaction energies [PA (blue circles), EC (green diamonds), SA (red triangles), and SE (orange squares)] versus distance for bacterial interactions: (**a**) $\Delta G_{132}^{LW}\left(\times 10^{-21}\right)$ where $d_{eq}=7.02\pm 0.00479\;\mathrm{nm}$ (*N* = 4), (**b**) $\Delta G_{13}^{LW}\left(\times 10^{-21}\right)$ where $d_{eq}=7.02\pm 0.0149\;\mathrm{nm}$ (*N* = 4), (**c**) $\Delta G_{132}^{AB}\left(\times 10^{-8}\right)$ where $d_{eq}=6.83\pm 0.00854\;\mathrm{nm}$ (*N* = 4) and (**d**) $\Delta G_{13}^{AB}\left(\times 10^{-8}\right)$ where $d_{eq}=6.81\pm 0.0193\;\mathrm{nm}$ (*N* = 4).

The predominately adhesive Gibbs interaction energies (i.e., $\Delta G^{LW}$ and $\Delta G^{AB}$) of each bacterium with superhydrophobic and superhydrophilic substrates with respect to distance are compared in Figure 3. $\Delta G^{LW}$ are provided in Figure 3a (superhydrophobic) and Figure 3b (superhydrophilic) while $\Delta G^{AB}$ are depicted in Figure 3c (superhydrophobic) and Figure 3d (superhydrophilic).

The trends for each plot demonstrate that adhesive bacteria-substrate interactions increase as the bacterium approaches the substrate. Comparing $\Delta G^{LW}$ and $\Delta G^{AB}$, the former has less magnitude indicating that $\Delta G^{AB}$ is the more influential adhesive bacteria-substrate interaction. Furthermore, $d_{eq}$ for each bacterium is in a narrow range of 6.81–7.02 nm regardless of bacteria shape or motility.

**Figure 4:**
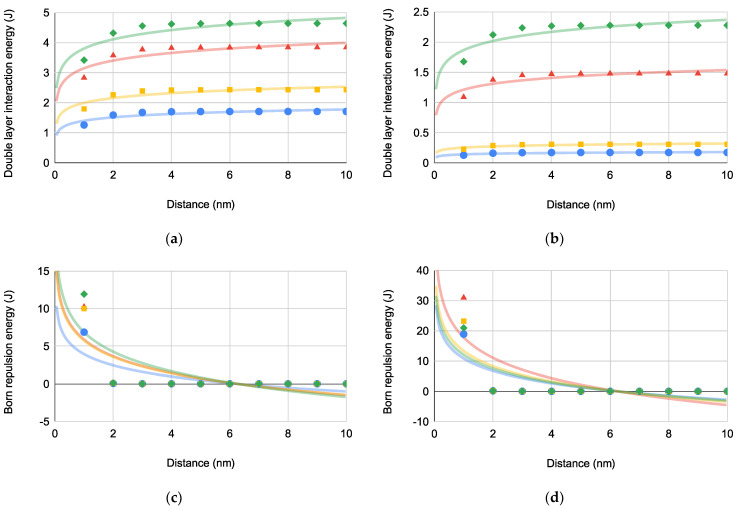
Bacterial Gibbs interaction energies [PA (blue circles), EC (green diamonds), SA (red triangles), and SE (orange squares)] versus distance for bacterial interactions: (**a**) $\Delta G_{132}^{EL}\left(\times 10^{-7}\right)$, (**b**) $\Delta G_{13}^{EL}\left(\times 10^{-7}\right)$, (**c**) $\Delta G_{132}^{Born}\left(\times 10^{-25}\right)$ where $d_{eq}=6.25\pm 0.00854\;\mathrm{nm}$ (*N* = 4), and (**d**) $\Delta G_{13}^{Born}\left(\times 10^{-25}\right)$ where $d_{eq}=6.24\pm 0.00957\;\mathrm{nm}$ (*N* = 4).

The interaction energies contributing most to bacteria-substrate repulsion (i.e., $\Delta G^{EL}$ and $\Delta G^{Born}$) are depicted in Figure 4 for both superhydrophobic and superhydrophilic substrates.

$\Delta G^{EL}$ is repulsive across all distances with a maximum approximating 6 nm for all bacteria-substrate interactions. $\Delta G^{Born}$ for bacteria-substrate interactions is increasingly repulsive when bacterial approach to the substrate is closer than $d_{eq}$ (6.24–6.25 nm). Also, $\Delta G^{EL}$ has a much larger magnitude than $\Delta G^{Born}$ indicating that $\Delta G^{EL}$ is the dominant repulsive bacteria-substrate interaction.

**Figure 5:**
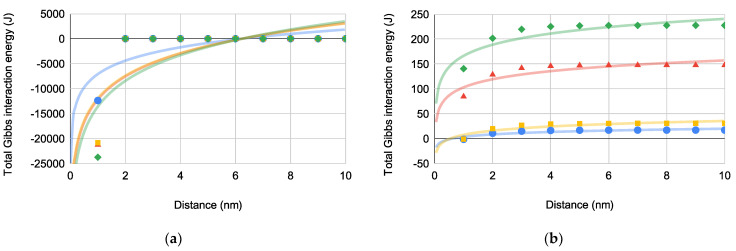
$\Delta G^{tot}\left(\times 10^{-9}\right)$ versus distance for bacterial interactions [PA (blue circles), EC (green diamonds), SA (red triangles), and SE (orange squares)] with (**a**) $\Delta G_{132}^{tot}$ where $d_{eq}=6.56\pm 0.0274\;\mathrm{nm}$ (*N* = 4) and (**b**) $\Delta G_{13}^{tot}$ where $d_{eq}$ approximates that of PBS for both PA and SE.

According to Equation (2), $\Delta G^{tot}$ may now be calculated by simple summation of $\Delta G^{LW}$, $\Delta G^{AB}$, $\Delta G^{EL}$, and $\Delta G^{Born}$ with respect to distance. $\Delta G_{132}^{tot}$ and $\Delta G_{13}^{tot}$ is depicted in Figure 5a,b, respectively.

Figure 5 differentiates $\Delta G^{tot}$ for bacterial-superhydrophobic substrate interactions (Figure 5a) and bacterial-superhydrophilic substrate interactions (Figure 5b). For superhydrophobic substrates, adhesive interactions become prominent as bacterial approach within 6.56 nm (i.e., $d_{eq}$). Across most approach distances for superhydrophilic substrates, repulsive interactions dominate with maxima around 6–7 nm. Therefore, bacterial response to superhydrophobic and superhydrophilic substrates is markedly different and is deserving of further discussion.

## 3. Discussion

### 3.1. Equilibrium and Bacterial Attachment

When a bacterium is suspended in an aqueous medium, the bacterium is functionally a colloid where interactions with a substrate (i.e., primary colonization) may be thermodynamically assessed for spontaneous interaction (i.e., attachment). By mitigating primary colonization as the initial step in biofilm formation, propagation and growth of bacteria in biofilms may be controlled thereby decreasing pathogenesis.

Thermodynamically, bacterial attachment to a substrate is a dynamic equilibrium involving both adhesion and repulsion. Primary colonization is the only reversible step in biofilm formation. If adhesive bacteria-substrate interactions are more significant than repulsive interactions, bacterial attachment is said to be spontaneous (i.e., thermodynamically favored) with a negative $\Delta G^{tot}$. However, when repulsive bacteria-substrate interactions dominate adhesive interactions, bacterial attachment is thermodynamically unfavored (i.e., non-spontaneous) yielding a positive $\Delta G^{tot}$. Examination of bacterial interaction with two substrates with very different surface energies [i.e., superhydrophobic (low surface energy) and superhydrophilic (high surface energy)] will help provide thermodynamic limits within which a bacterium interacts with a substrate. To model the equilibrium of bacterial attachment and thereby thermodynamically address the primary colonization mechanism, extended DLVO theory may be applied to aqueous bacterial suspensions and their interaction with substrates. For our purposes, we can use extended DLVO theory to determine (1) the equilibrium distance ($d_{eq}$) where adhesive forces are equivalent to repulsive interactions and (2) the contribution of the individual adhesive and repulsive components to the dynamic equilibrium of bacterial attachment leading toward primary colonization.

Since extended DLVO theory is based in part on the distance of approach of a colloid (i.e., bacterium) to a substrate, $d_{eq}$ is a critical determinant with respect to the equilibrium of bacterial attachment to a substrate. At an equilibrium approach distance (i.e., $d_{eq}$) where ΔG=0 J, adhesive and repulsive bacterial-substrate interactions are equivalent, resulting in a determinable $d_{eq}$ (Table 3).

We found that $d_{eq}$ for bacteria-bacteria interactions was 2.80 nm whereupon nonpolar cell aggregation would be favored (i.e., spontaneous) when $d<d_{eq}$ else the cells would remain suspended in aqueous solution. Considering superhydrophobic and superhydrophilic substrates, adhesive bacteria-substrate interactions predominate when $d<d_{eq}$ for $\Delta G^{LW}$ and $\Delta G^{AB}$ and when $d_{eq}<d$ with regard to $\Delta G^{Born}$. When repulsive bacterial-substrate interactions with superhydrophobic and superhydrophilic substrates dominate, the bacteria remain suspended in aqueous solution as described by $\Delta G^{LW}$, $\Delta G^{AB}$, and $\Delta G^{EL}$ when $d_{eq}<d$ and also for $\Delta G^{Born}$ when $d<d_{eq}$.

Each of the isolable components from extended DLVO theory (e.g., $\Delta G^{LW}$, $\Delta G^{AB}$, $\Delta G^{EL}$, and $\Delta G^{Born}$) provide a non-equivalent portion of the mechanistic story for bacterial-substrate primary colonization interactions and attachment. To that end, Table 4 summarizes the percent contribution of each component energy at equilibrium (i.e., $\Delta G^{tot}=0\;J$) toward the total thermodynamic picture of bacteria-substrate interaction.

Examination of the percent contribution illustrate that both $\Delta G^{LW}$ (adhesive) and $\Delta G^{Born}$ (repulsive) do not significantly contribute to the mechanism for bacterial attachment to either superhydrophobic or superhydrophilic substrates. For bacterial attachment to superhydrophobic substrates, $\Delta G^{AB}$ (adhesive) and $\Delta G^{EL}$ (repulsive) are the major contributors. Interestingly, only the repulsive $\Delta G^{EL}$ significantly affects bacterial attachment to superhydrophilic substrates. Thus, bacterial-substrate attachment mechanisms are very complex and diverse but can be thermodynamically assessed to determine both favorable (i.e., spontaneous) and unfavorable (i.e., non-spontaneous) bacteria-substrate interactions involving sphere (i.e., coccus)-plane and spherocylindrical (i.e., bacillus)-plane geometric interactions.

### 3.2. Short Range Interactions and Bacterial Attachment

Traditionally, short range interactions have been Coulombically defined relating interacting particle charges with respect to distance. The interaction distances are very close resulting in (1) Lifshitz-van der Waals adhesive interactions based on induced dipolar forces caused by the approach of one species’ electron cloud to another’s and (2) Born repulsive interactions involving the repulsion of two ionic species in close proximity. Both adhesive and repulsive interactions have a defined $d_{eq}$ at which point the interaction increases significantly as $d<d_{eq}$.

#### 3.2.1. Lifshitz-Van der Waals Adhesive Interactions

The effect of the non-polar adhesive interactions on $\Delta G^{LW}$ may be determined via surface energies or extended DLVO theory. By using each bacterium’s unique Hamaker constant (from surface energies) and its relation to $\Delta G^{LW}$, bacterial radius, and approach distance, we were able to determine the nonpolar equilibrium interaction distance ($d_{eq}^{LW}$). For both superhydrophobic and superhydrophilic substrates, $d_{eq}^{LW}$ is 3.58 nm. Yet the extended DLVO theory when applied to both superhydrophobic and superhydrophilic substrates and similarly accounting for bacterial radius and approach distance yielded a $d_{eq}^{LW}$ of 7.02 nm when considering the bacterial surface areas. We posit that the latter $d_{eq}^{LW}$ is likely the actual value for the nonpolar adhesive interactions. Based on our findings, substrate polarity does not affect $d_{eq}^{LW}$ for bacterium-substrate nonpolar interactions. More intriguingly, $d_{eq}^{LW}$ is independent of bacterial species, size, and shape. Therefore, $d_{eq}^{LW}$ may be an intrinsic value for the examined bacterial interactions with a substrate.

Furthermore, $\Delta G^{LW}$ is relatively small in magnitude for bacterial interactions with superhydrophobic and superhydrophilic substrates ($-10^{-21}\;J$ to $-10^{-20}\;J$ per bacterium). The magnitude of $\Delta G^{LW}$ is slightly more pronounced for the superhydrophilic substrates due to the comparatively larger nonpolar surface energy than that of the superhydrophobic substrate. Therefore, $\Delta G^{LW}$ is a relatively minor contributor to the overall attachment thermodynamics.

Three of four bacteria examined showed a spontaneous trend for adhesion to a superhydrophobic substrate while all bacteria exhibited a non-spontaneous adhesive trend for superhydrophilic substrates indicating that the bacteria would thermodynamically prefer aqueous suspension to penetration into the hydration layer.

#### 3.2.2. Born Repulsive Interactions

Considering the interfacial interaction of an ionic bacterial surface with either a superhydrophobic or superhydrophilic substrate, Born repulsive interactions are possible when in close proximity. Neither substrate is ionically charged given the nonpolar nature of a superhydrophobic substrate or the hydration layer associated with a superhydrophilic substrate; therefore, the bacteria-substrate interaction is an ion-dipole interaction. Since the interaction does not involve ion-ion interactions, the magnitude of repulsion should be diminutive. Thus, we find that $\Delta G^{Born}$ for each examined bacterium with both superhydrophobic and superhydrophilic substrates becomes non-spontaneous with very small magnitudes on the order of $+10^{-24}\;J$ per bacterium leading to the conclusion that $\Delta G^{Born}$ is also a relatively minor contributor to the overall attachment thermodynamics.

When evaluating $d_{eq}^{Born}$, substrate polarity and bacterial species, size, and shape seem to not affect $d_{eq}^{Born}$ approximating 6.24 nm. As with $d_{eq}^{LW}$, $d_{eq}^{Born}$ seems to be an intrinsic value of the examined bacteria-substrate interactions. Finally, PA is least affected by Born repulsion regardless of substrate examined; however, no other significant trend is observed.

### 3.3. Long Range Interactions and Bacterial Attachment

Long range interactions in simple ion-counterion systems are easily modeled, but polyionic systems (e.g., bacteria-substrate interactions) are significantly more complex. Bacteria function as large colloids wherein the many peripheral ions effectively yield a species that is very charged/ionic. The aforementioned summation of intermolecular forces due to the multitude of ions on a bacterium result in an adhesive acid-base (e.g., $\Delta G^{AB}$) and repulsive electrostatic double layer (e.g., $\Delta G^{EL}$) interactions that are orders of magnitude larger than their short range counterparts (e.g., $\Delta G^{LW}$ and $\Delta G^{Born}$). Comprised of electron acceptor/donor interactions between two species, a plot of $\Delta G^{AB}$ versus distance yields a defined $d_{eq}^{AB}$ wherein the spontaneity of interaction becomes much more pronounced. Unlike all of the other component energies in the extended DLVO theory, $\Delta G^{EL}$ is consistently repulsive (i.e., non-spontaneous) across most examined distances resulting in a very small $d_{eq}^{EL}$, if any.

#### 3.3.1. Acid-Base Adhesive Interactions

In general, $\Delta G^{AB}$ is much more pronounced for the superhydrophobic substrate relative to the superhydrophilic substrate (i.e., 100 times greater). The superhydrophobic substrate has a moderate surface energy acid component ($\gamma_{2}^{+}$) which is able to strongly interact with the large base component of the bacteria ($\gamma_{1}^{-}$); essentially, there is no appreciable base component for superhydrophobic substrates ($\gamma_{2}^{-}$). Given the hydration layer associated with the superhydrophilic substrate, the equivalent acid ($\gamma_{3}^{+}$) and base ($\gamma_{3}^{-}$) components of water/substrate produce no appreciable interaction with the bacterium’s base component ($\gamma_{1}^{-}$) likely yielding a preference for the bacterium to remain suspended in the aqueous solvent. Furthermore when $d<d_{eq}^{AB}$, $\Delta G^{AB}$ is orders of magnitude ($-10^{-8}\;J$ to $-10^{-6}\;J$ per bacterium) more spontaneous than the short range $\Delta G^{LW}$ and is thus much more influential in attachment of a bacterium to a substrate regardless of polarity. As observed for $d_{eq}^{LW}$ and $d_{eq}^{Born}$, $d_{eq}^{AB}$ seems to be intrinsic to the bacteria-substrate interaction with a value of 6.82 nm and is independent of substrate polarity and bacteria species, size, and shape.

Again, PA is closest to the defined equilibrium value ($\Delta G^{tot}=0\;J$) for both substrates across most examined distances. In comparison to both $\Delta G^{LW}$ and $\Delta G^{Born}$ for the superhydrophobic substrate, $\Delta G^{AB}$ has the largest magnitude and therefore dominates $\Delta G^{tot}$ as shown in Figure 5a.

#### 3.3.2. Electrostatic Double Layer Repulsive Interactions

As the dominate repulsive interaction, $\Delta G^{EL}$ is non-spontaneous across all distances for most bacteria and both substrates. $\Delta G^{EL}$ for both superhydrophobic and superhydrophilic substrates is many orders of magnitude larger (+$10^{-8}\;J$ to +$10^{-7}\;J$ per bacterium) than either $\Delta G^{LW}$ or $\Delta G^{Born}$, even at small distances. For superhydrophilic substrates, $\Delta G^{EL}$ dominates the bacteria-substrate interaction (Figure 5b) indicating that the bacteria would prefer suspension in the aqueous solvent to penetration of the hydration layer. No $d_{eq}^{EL}$ is observed for $\Delta G^{EL}$; however, a maximum $\Delta G^{EL}$ is reached between 6–7 nm for all bacteria with all substrates. As with $\Delta G^{Born}$, $\Delta G^{EL}$ for PA is the least non-spontaneous of the examined bacteria.

### 3.4. Effect of Bacterial Motility

Comparing the attachment of motile bacteria (PA and EC) with non-motile bacteria (SA and SE) onto both superhydrophobic and superhydrophilic substrates, bacterial motility seems to have little effect on attachment. If bacterial motility was a significant contributor to the attachment thermodynamics with specific attention to $\Delta G^{LW}$, $\Delta G^{AB}$ and $\Delta G^{Born}$, we would expect variable $d_{eq}$ for each bacterium; however, this is not the case. For example, the nonmotile bacteria have a slightly more spontaneous $\Delta G^{LW}$ (nonpolar interaction) with the superhydrophilic substrate relative to the motile bacteria; yet, $d_{eq}$ remains relatively constant for all examined bacteria indicating a propensity to not penetrate the hydration layer. Interestingly, the logarithmic trendlines yield a near identical $d_{eq}$ at approximately 7 nm for each bacterium-substrate interaction energy component (i.e., $\Delta G^{LW}$, $\Delta G^{AB}$, and $\Delta G^{Born}$). $d_{eq}$ seems to be independent of bacterial species, bacterial motility, species, size, and shape as well as substrate polarity. Thus, $d_{eq}$ of a bacterium with a substrate may be an intrinsic property describing the polarizability of a bacterium-substrate interaction. Furthermore, $\Delta G^{EL}$ seems to yield a maximum repulsive interaction at values approximating $d_{eq}$.

When comparing each of the extended DLVO component energies with bacterial motility, the motile bacteria have the largest and smallest energies with the exception of $\Delta G_{13}^{LW}$ and $\Delta G_{13}^{Born}$ (superhydrophilic substrate) which are both very small relative to $\Delta G_{13}^{AB}$ and $\Delta G_{13}^{EL}$. The nonmotile bacteria are more affected by $\Delta G^{Born}$ than the motile bacteria which have a larger surface area, but again, $\Delta G^{Born}$ is less influential to the overall attachment mechanism. Furthermore, surface energy analysis seems to indicate that the attachment mechanism onto superhydrophobic substrates are thermodynamically controlled where bacterial motility may have a minimal contribution to adsorption thereto. Therefore, we must conclude from our data that bacterial motility likely has no appreciable contribution to the thermodynamics of bacterial attachment to a substrate.

### 3.5. Significance of Bacterial Attachment Equilibria

Based on a comparison of magnitudes for each of the extended DLVO energy components (i.e., $\Delta G^{LW}$, $\Delta G^{AB}$, $\Delta G^{EL}$, and $\Delta G^{Born}$) for bacterial attachment to either superhydrophobic or superhydrophilic substrates, $\Delta G^{AB}$ (adhesive) and $\Delta G^{EL}$ (repulsive) are the major contributors. Therefore, we can simplify Equation (2) to the following [Equation (9)]:

(9) $$\Delta G^{tot}\approx \Delta G^{AB}+\Delta G^{EL}$$

While Equation (9) is postulated to generally apply to bacterium-substrate interactions, specialized substrates (e.g., superhydrophobic and superhydrophilic) seem to trend in such a way that the thermodynamics of attachment can be uniquely quantified. Considering superhydrophobic substrates, $\Delta G^{EL}$ is approximately three times larger than $\Delta G^{AB}$ at $d_{eq}$ making $\Delta G_{132}^{tot}$ quantifiable from either $\Delta G_{132}^{AB}$ or $\Delta G_{132}^{EL}$ [Equations (10) and (11)].

(10) $$\Delta G_{132}^{tot}\approx \frac{4}{3}\cdot \Delta G_{132}^{EL}$$

(11) $$\Delta G_{132}^{tot}\approx 4\cdot \Delta G_{132}^{AB}$$

For superhydrophilic substrates, only the repulsive $\Delta G^{EL}$ significantly affects bacterial attachment to superhydrophilic substrates resulting in a $\Delta G^{tot}$ that approximates $\Delta G^{EL}$ [Equation (12)] which is non-spontaneous for most approach distances.

(12) $$\Delta G_{13}^{tot}\approx \Delta G_{13}^{EL}$$

Based on our findings, the thermodynamics of bacteria-substrate adhesion are independent of substrate polarity as well as bacterial motility, species, size, and shape leading us to conclude that the bacterial-substrate adhesion is mitigated by only those underlying factors upon which $\Delta G^{AB}$ and $\Delta G^{EL}$ are based. $\Delta G^{AB}$ is dependent on the approach distance and is directly related to the interfacial interaction via the summation of the contact angles of the solvent (i.e., $cos\;\theta_{13}$ and $cos\;\theta_{23}$, respectively) with both the bacterium and substrate. While also dependent on the approach distance, $\Delta G^{EL}$ is also dependent on the zeta potentials of both bacterium and substrate (i.e., $\zeta_{1}$ and $\zeta_{2}$, respectively).

**Figure 6:**
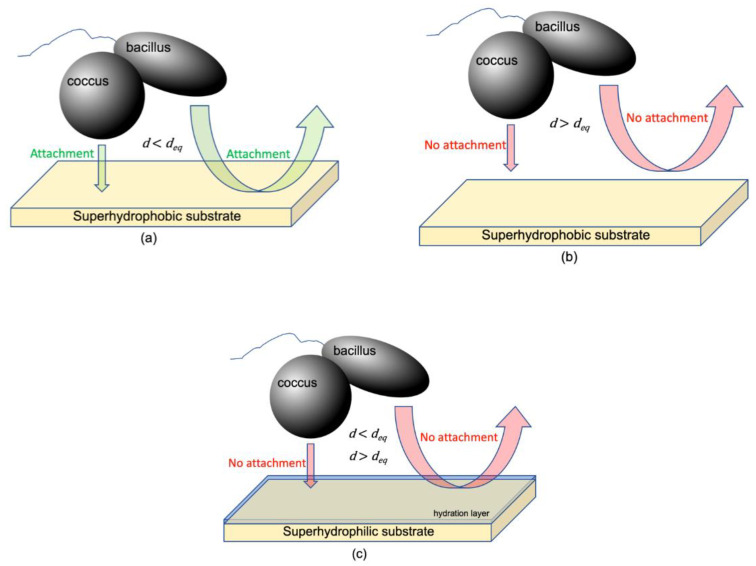
Distance dependence of bacterial attachment for (**a**) superhydrophobic substrates where $d\leq d_{eq}$; (**b**) superhydrophobic substrates where $d>d_{eq}$; and (**c**) superhydrophilic substrates where $d\leq d_{eq}$ and $d>d_{eq}$.

Both $\Delta G^{EL}$ and $\Delta G^{EL}$ are relatively easy to experimentally determine yielding a facile way to evaluate bacteria-substrate interactions to inhibit biofilm formation and pathogenesis. In other words, the adhesive-repulsive equilibrium determines bacterial attachment to superhydrophobic substrates while bacterial attachment to superhydrophilic substrates is most affected by repulsive forces and seems to be non-spontaneous at all distances (Figure 6).

Using this research should allow substrates to be designed that will inhibit biofilm formation at the initial and reversible primary colonization step thereby inhibiting the large scale, protected biofilm growth that produces most pathogenic bacteria.

Attachment of bacteria to a substrate as described herein seems to yield one major caveat originating from the calculated values of $d_{eq}$. The small value of $d_{eq}$ seems to indicate that bacteria are most stable when suspended in aqueous media unless bacteria penetrate $d_{eq}$, so how does primary colonization occur in the first place? The underlying assumption in this modeling research is that the bacterium itself is what adheres to the substrate; however, our research shows that another thermodynamic interaction must occur for primary colonization to be spontaneous.

## 4. Materials and Methods

### 4.1. Gibbs Interaction Energies for Homogeneous Substrates

Assuming that each substrate is both smooth and homogeneous, we determined the Gibbs interaction energies via surface energy measurements and extended DLVO theory.

#### 4.1.1. Determination from Surface Energies

Surface energy measurements relate to total (i.e., summative) Gibbs interaction energy ($\Delta G^{tot}$) and may be separated into the nonpolar (i.e., Lifshitz-van der Waals, $\Delta G^{LW}$) and polar (i.e., acid-base, $\Delta G^{AB}$) components [Equations (5) and (7)].

To determine a bacterium’s nonpolar interaction with superhydrophobic substrates in water, Equation (13) was used [33]:

(13) $$\Delta G_{132}^{LW}=\gamma_{12}^{LW}-\gamma_{13}^{LW}-\gamma_{23}^{LW}$$

where $\gamma_{12}^{LW}$ indicates the Lifshitz-van der Waals nonpolar interaction surface energy between the bacterium and substrate, $\gamma_{13}^{LW}$ indicates the Lifshitz-van der Waals nonpolar interaction surface energy between the bacterium and water, and $\gamma_{23}^{LW}$ indicates the Lifshitz-van der Waals nonpolar interaction surface energy between the substrate and water. Each of the aforementioned nonpolar interactions were calculated based on Equation (14) [33]:

(14) $$\gamma_{ij}^{LW}=\gamma_{i}^{LW}+\gamma_{j}^{LW}-2\sqrt{\gamma_{i}^{LW}\gamma_{j}^{LW}}$$

where the subscripts (*i* and *j*) indicate any two distinct materials.

Since superhydrophilic materials are assumed to have a closely associated surface layer of water with similar surface energy properties to water, the determination of a bacterium’s nonpolar interaction therewith is simpler as shown in Equation (15) [33]:

(15) $$\Delta G_{13}^{LW}=\gamma_{13}^{LW}-\gamma_{1}^{LW}-\gamma_{3}^{LW}$$

where $\gamma_{1}^{LW}$ is the nonpolar surface energy component of the bacterium and $\gamma_{3}^{LW}$ is that of the substrate (i.e., water).

From Equations (3) and (16), Hamaker constants ($H_{i}$) were determined for each bacterium as well as the interaction of water-dispersed bacteria with the superhydrophobic or superhydrophilic substrates:

(16) $$H_{i}=\frac{\Delta G_{i}^{LW}r_{1}}{6d}$$

where *d* is the distance between bacterium and substrate, $r_{1}$ is the bacterial radius, and the subscript (i) indicates the bacteria-water-substrate interaction for either superhydrophobic or superhydrophilic substrates [33]. Assuming a sphere-plate interaction, Equations (16) account for the geometry of each bacterium [i.e., sphere for *S. aureus* (SA) and *S. epidermidis* (SE) or spherocylindrical for *P. aeruginosa* (PA) and *E. coli* (EC)] interacting with the substrate.

Using similar mathematical treatments, ${\Delta G}^{\mathrm{tot}}$ of a bacterium dispersed in water with a superhydrophobic or superhydrophilic substrate may be determined via Equations (7) and (9), respectively.

Knowing that the surface energetic approach for determining the Gibbs interaction energy of a bacterium dispersed in water with a substrate does not account for either Born repulsion ($\Delta G^{Born}$) or electrostatic double layer repulsion ($\Delta G^{EL}$), we determined $\Delta G^{AB}$ by the difference in the $\Delta G^{tot}$ and $\Delta G^{LW}$ for both superhydrophobic or superhydrophilic substrates [Equations (17) and (18), respectively]:

(17) $$\Delta G_{132}^{AB}=\Delta G_{132}^{tot}-\Delta G_{132}^{LW}$$

(18) $$\Delta G_{13}^{AB}=\Delta G_{13}^{tot}-\Delta G_{13}^{LW}$$

For comparison with Equation (19), we used an analogue of Equations (8) and (14) to determine $\Delta G^{AB}$ for superhydrophilic substrates [i.e., Equation (19)]:

(19) $$\Delta G_{13}^{AB}=\gamma_{13}^{AB}-\gamma_{1}^{AB}-\gamma_{3}^{AB}$$

Table 5 provides all surface energy data and sources thereof used to perform the surface energy numerical experiments.

#### 4.1.2. Determination from Extended DLVO Theory

The ${\Delta G}^{\mathrm{tot}}$ may be calculated via the extended DLVO theory according to Equation (2); however, the component interaction energies (e.g., $\Delta G^{LW}$, $\Delta G^{AB}$, $\Delta G^{EL}$, and $\Delta G^{Born}$) need further examination, especially as related to the individual geometries of the bacteria. The following approach is largely defined by Bradford et al., where $\Delta G^{LW}$ may be determined from Equation (20) [17]:

(20) $$\Delta G^{LW}=-\frac{H_{132}r_{1}}{6d}{\left(1+\frac{14d}{\lambda }\right)}^{-1}$$

where λ=100 nm and is a characteristic wavelength, $r_{1}$ is the radius of the bacterium, and *d* is the distance between bacterium and substrate [40]. The Hamaker constant for the bacterium-water-substrate interfacial interaction ($H_{132}$) is determined via Equation (21) [41]:

(21) $$H_{132}=\left(\sqrt{H_{1}}-\sqrt{H_{3}}\right)\left(\sqrt{H_{2}}-\sqrt{H_{3}}\right)$$

where $H_{3}=3.7\times 10^{-20}\;J$ and the value of $H_{2}$ for the superhydrophobic substrate is calculated based on Equation (22) in which $d_{o}$ is the value of closest approach taken to be 0.157 nm [42]:

(22) $$H_{2}=24\pi \gamma_{2}^{LW}{d_{o}}^{2}$$

Since the equilibrium distance ($d_{eq}$) separating a bacterium and the substrate is dependent on the nature of the bacterium, substrate, and dispersing solution, the bacterial Hamaker constants ($H_{1}$) were determined graphically with respect to distance (d) to the substrate using Equation (3) [33].

The graphically determined value of $H_{1}$ for *EC* is comparable to the average value determined by Janjaroen et al. [14].

$\Delta G^{AB}$ is determined using Equation (23) [42]:

(23) $$\Delta G^{AB}=2\pi r_{1}\lambda_{AB}\Delta G_{d=d_{o}}^{AB}exp\left(\frac{d_{o}-d}{\lambda_{AB}}\right)$$

where $\lambda_{AB}$ (1 nm) is a characteristic decay length of acid-base interactions in water [41] and $\Delta G_{d=d_{o}}^{AB}$ is the Lewis acid-base interaction energy per area between two surfaces when $d_{o}=d$ calculated via Equations (24) and (25) [43,44]:

(24) $$\Delta G_{d=d_{o}}^{AB}=-\frac{K}{2\pi d_{o}\lambda_{AB}}$$

(25) $$log\;K=-7.0\left(\frac{cos\;\theta_{13}+cos\;\theta_{23}}{2}\right)-18.0$$

$\Delta G^{EL}$ is calculated via Equation (26) [45]:

(26) $$\Delta G^{EL}=\pi \varepsilon_{3}r_{1}\left({\zeta_{1}}^{2}+{\zeta_{2}}^{2}\right)\left\{\frac{2\zeta_{1}\zeta_{2}}{{\zeta_{1}}^{2}+{\zeta_{2}}^{2}}\;ln\;\left[\frac{1+exp\left(-\kappa d\right)}{1-exp\left(-\kappa d\right)}\right]+ln\;\left[1-exp\left(-\kappa d\right)\right]\right\}$$

where $\varepsilon_{3}$ is the dielectric constant of water, $\zeta_{1}$ and $\zeta_{2}$ are the respective zeta potentials of bacterium and substrate, and κ is the inverse Debye length.

To account for $\Delta G^{Born}$, Equation (27) is utilized:

(27) $$\Delta G^{Born}=\frac{H_{132}{\sigma_{c}}^{6}}{7560}\left[\frac{8r_{1}+d}{{\left(2r_{1}+7\right)}^{7}}+\frac{6r_{1}-d}{d^{7}}\right]$$

where $\sigma_{c}$ (0.26 nm) and is the collision diameter which would achieve a primary minimum depth of 0.157 nm [17,46].

Table 6 provides all relevant constants and sources thereof while Table 7 provides the aggregated data to perform the extended DLVO numerical experiments.

### 4.2. Numerical Experiments

Our experimentation utilizes literature components to formulate Gibbs interaction energies determined via surface energy analyses and extended DLVO theory. In order to standardize the data, several experimental parameters were established, some of which were based on fundamental assumptions.

Our surface energy measurements [e.g., Lifshitz-van der Waals nonpolar component ($\gamma^{LW}$), polar acid-base component ($\gamma^{AB}$), acid component ($\gamma^{+}$), base component ($\gamma^{-}$), and total surface energy ($\gamma^{tot}$)] were assumed to be temperature independent across the fairly narrow range between room temperature (20–25 °C) and physiological temperature (37 °C). Volatility of the solvents used for surface energy calculations would provide the most significant source of error with variable temperatures. The solvents from which the measurements were obtained are not very volatile and would persist for the time frames during which the measurements were taken.

For the measurements related to extended DLVO theory, we ensure all measurements were obtained in a physiological mimic solution, phosphate buffer solution (PBS), with neutral or physiological pH values. Since PBS is buffered, pH is relatively constant across a wide range of additional acids or bases. Therefore, dilute bacterial dispersions would not significantly alter pH nor be affected by PBS. Furthermore, the bacterial zeta potentials ($\zeta_{1}$) were all obtained in PBS having a pH between 7.0 and 7.4. The inverse Debye length (κ), used in the measurement of $\Delta G^{EL}$, was determined to be 0.75 nm in 1× PBS [48].

Superhydrophilic substrates exposed to an aqueous medium are assumed to yield surface properties and chemistry closely resembling those of pure water which is consistent with the experimentally observed wetting behavior of such substrates (e.g., underwater oil contact angles) [34,35,36,55]. The aforementioned experimental observation seems to indicate that ions would be largely excluded from the hydration layer associated with the substrate [35,56]. Thus, the surface energies, zeta potentials, and other related data for superhydrophilic substrates are assumed to be identical to water.

## 5. Conclusions

We purposed to mathematically model bacterial interactions with superhydrophobic and superhydrophilic substrates to determine the thermodynamic components of bacteria-substrate interactions leading to attachment and primary colonization. Assuming colloidal behavior for the bacteria, we used extended DLVO theory to assess four thermodynamic components of bacterial attachment to a substrate: Lifshitz-van der Waals nonpolar adhesion ($\Delta G^{LW}$), acid-base polar adhesion ($\Delta G^{AB}$), electrostatic double layer repulsion ($\Delta G^{EL}$), and Born repulsion ($\Delta G^{Born}$). The equilibrium distance ($d_{eq}$) was determined to be 6–7 nm for $\Delta G^{LW}$, $\Delta G^{AB}$, and $\Delta G^{Born}$ and is independent of substrate polarity and bacterial motility, species, size, and shape. Thus, $d_{eq}$ may be intrinsic to bacteria-substrate interactions. Both $\Delta G^{LW}$ and $\Delta G^{Born}$ were determined to be minor contributors to the overall bacteria-substrate interaction for both substrates ($\Delta G^{tot}$). Therefore, we were able to simplify the extended DLVO theory illustrating primary dependence of $\Delta G^{tot}$ which assesses the thermodynamic favorability of bacterial-substrate interactions on $\Delta G^{AB}$ and $\Delta G^{EL}$ for bacteria-substrate interactions. For superhydrophobic substrates, both $\Delta G^{AB}$ and $\Delta G^{EL}$ regulate the thermodynamics of bacteria-substrate interactions with adhesion predominating when the approach distance is less than the equilibrium distance ($d<d_{eq}$). Due to the hydration layer associated with superhydrophilic substrates, bacteria seem to thermodynamically prefer suspension to adhesion evidenced by the predominance of the repulsive $\Delta G^{EL}$. Thus, superhydrophilic substrates should be most resistant to primary colonization and bacterial attachment. Unfortunately, the model herein also illustrates that the colloidal treatment of bacteria as a whole to determine the bacteria-substrate attachment mechanism needs further evaluation. However, the model herein could assist scientists in the design of substrates that are resistant to primary colonization which often produces a mature biofilm that becomes a major contributor to pathogenic bacteria.

## Figures and Tables

**Table 1:** Numerical experimental surface energy results for bacteria (PA, SA, SE, and EC) with superhydrophobic substrates where the subscripted 132 indicates bacteria dispersed in water interacting with a superhydrophobic substrate.

Surface Energy	PA	SA	SE	EC
$\Delta G_{132}^{LW},\;\mathrm{mJ}/m^{2}$	4.87	6.43	2.25	−1.62
$\Delta G_{132}^{AB},\;\mathrm{mJ}/m^{2}$	−33.39	−32.77	−21.72	−14.70
$\Delta G_{132}^{tot},\;\mathrm{mJ}/m^{2}$	−28.52	−26.34	−19.47	−16.32
$\Delta G_{132}^{tot}\cdot A_{s},\;J$	$-1.31\times 10^{-13}$	$-8.28\times 10^{-14}$	$-4.96\times 10^{-14}$	$-1.03\times 10^{-13}$

**Table 2:** Numerical experimental surface energy results for bacteria (PA, SA, SE, and EC) with superhydrophilic substrates where the subscripted 13 indicates bacteria dispersed in water interacting with a superhydrophilic substrate with surface energies equivalent to water.

Surface Energy	PA	SA	SE	EC
$\Delta G_{13}^{LW},\;\mathrm{mJ}/m^{2}$	−55.10	−58.79	−48.92	−39.77
$\Delta G_{13}^{AB,sum},\;\mathrm{mJ}/m^{2}$	−86.95	−89.20	−91.14	−97.82
$\Delta G_{13}^{AB,calc},\;\mathrm{mJ}/m^{2}$	−102.89	−104.54	−95.42	−95.08
$\Delta G_{13}^{tot,calc},\;\mathrm{mJ}/m^{2}$	−142.05	−147.99	−140.06	−137.59
$\Delta G_{13}^{tot,sum},\;\mathrm{mJ}/m^{2}$	−158.00	−163.32	−144.34	−134.85
$\Delta G_{13}^{tot,calc}\cdot A_{s},\;J$	$-6.53\times 10^{-13}$	$-4.65\times 10^{-13}$	$-3.56\times 10^{-13}$	$-8.64\times 10^{-13}$
$\Delta G_{13}^{tot,sum}\cdot A_{s},\;J$	$-7.26\times 10^{-13}$	$-5.13\times 10^{-13}$	$-3.67\times 10^{-13}$	$-8.47\times 10^{-13}$
Percent Difference	11.23	10.36	3.06	1.99

**Table 3:** $d_{eq}$ and effect of bacterial approach to a defined substrate accounting for bacteria-substrate interaction geometries and bacterial surface area.

Substrate	Interaction Energy	$d_{eq}$, nm	$d<d_{eq}$	$d_{eq}<d$
Bacteria	$\Delta G^{LW}$	2.80 ± 0.00479 3.58 ± 0.00267	aggregation	suspension
Superhydrophobic	$\Delta G^{LW}$	7.02 ± 0.00479	adhesion	suspension
	$\Delta G^{AB}$	6.83 ± 0.00854	adhesion	suspension
	$\Delta G^{EL}$	---	---	suspension
	$\Delta G^{Born}$	6.25 ± 0.00854	suspension	adhesion
Superhydrophilic	$\Delta G^{LW}$	7.02 ± 0.0149	adhesion	suspension
	$\Delta G^{AB}$	6.81 ± 0.0193	adhesion	suspension
	$\Delta G^{EL}$	---	---	suspension
	$\Delta G^{Born}$	6.24 ± 0.00957	suspension	adhesion

**Table 4:** Percent contribution to $\Delta G^{tot}$ of extended DLVO components ($\Delta G^{LW}$, $\Delta G^{AB}$, $\Delta G^{EL}$, and $\Delta G^{Born}$) for bacterial (PA, SA, SE, and EC) interactions with superhydrophobic and superhydrophilic substrates at $d_{eq}$ where negative percentages illustrate adhesive (i.e., spontaneous) interactions and positive percentages illustrate repulsive (i.e., non-spontaneous) interactions.

Substrate	Interaction Energy	PA	SA	SE	EC
Superhydrophobic	$\Delta G^{LW}$, %	$-7.11\times 10^{-14}$	$-4.20\times 10^{-14}$	$-8.00\times 10^{-14}$	$-3.96\times 10^{-14}$
	$\Delta G^{AB}$, %	−51.15	−34.94	−66.36	−31.51
	$\Delta G^{EL}$, %	151.15	134.94	166.36	131.51
	$\Delta G^{Born}$, %	$1.45\times 10^{-21}$	$8.58\times 10^{-22}$	$1.63\times 10^{-21}$	$8.08\times 10^{-22}$
Superhydrophilic	$\Delta G^{LW}$, %	$-1.30\times 10^{-12}$	$-2.45\times 10^{-13}$	$-8.88\times 10^{-13}$	$-1.08\times 10^{-13}$
	$\Delta G^{AB}$, %	−0.383	−0.0758	−0.357	−0.0550
	$\Delta G^{EL}$, %	100.38	100.08	100.36	100.05
	$\Delta G^{Born}$, %	$2.65\times 10^{-20}$	$5.01\times 10^{-21}$	$1.81\times 10^{-20}$	$2.20\times 10^{-21}$

**Table 5:** Surface energy data used to determine Gibbs interaction energies where $\gamma^{tot}$ is the total (i.e., summative) surface energy, $\gamma^{LW}$ is the Lifshitz-van der Waals (i.e., nonpolar) surface energy component, $\gamma^{AB}$ is the acid-base (i.e., polar) surface energy component, $\gamma^{+}$ is the acid surface energy component, and $\gamma^{-}$ is the base surface energy component.

Surface Energy	PA [18]	SA [18]	SE [37]	EC [38]	Superhydrophobic ^a^	Superhydrophilic ^b^
$\gamma^{tot}$	39.26	43.91	55.36	55.88	8.63	72.8
$\gamma^{LW}$	34.82	39.63	27.44	18.14	7.25	21.8
$\gamma^{AB}$	4.44	4.29	27.92	37.74	1.38	52
$\gamma^{+}$	0.089	0.066	4.11	7.83	9.84	25.5
$\gamma^{-}$	69.07	73.54	48.96	47.43	0.05	25.5

^a^ calculated from data provided by Sun et al. [39]. ^b^ assumed to be identical to pure water.

**Table 6:** Constants used to determine Gibbs interaction energies from extended DLVO theory.

Description	Value
$\mathrm{Dielectric}\;\mathrm{constant}\;\mathrm{of}\;H_{2}O\;\mathrm{at}\;37\;{}^{\circ}C\;(\varepsilon_{3}$$,\;C^{2}N^{-1}m^{-2}$)	74.15 [47]
Characteristic wavelength (λ, nm)	100 [40]
$\mathrm{Correlation}/\mathrm{decay}\;\mathrm{length}\;\mathrm{in}\;H_{2}O\;(\lambda_{AB}$, nm)	1 [41]
$\mathrm{Value}\;\mathrm{of}\;\mathrm{closest}\;\mathrm{approach}\;(d_{o}$, nm)	0.157 [42]
$\mathrm{Collision}\;\mathrm{diameter}\;(\sigma_{c}$, nm)	0.26 [17]
Debye length in 1× PBS (κ, nm)	0.75 [48]
$\mathrm{Hamaker}\;\mathrm{constant}\;\mathrm{of}\;H_{2}O\;(A_{3}$, J)	$3.70\times 10^{-20}$ [41]

**Table 7:** Additional data used to determine Gibbs interaction energies via extended DLVO theory.

Data	PA	SA	SE	EC	Superhydrophobic	Superhydrophilic
$r_{1}$, μm	0.325 [49]	0.50 [50]	0.45 [50]	0.50 [51]		
$A_{s}$, ${\mu m}^{2}$	4.595	3.142	2.545	6.283		
$H_{i}$, J	$3.78\times 10^{-21}$	$4.30\times 10^{-21}$	$2.98\times 10^{-21}$	$1.97\times 10^{-21}$	$1.60\times 10^{-20}$ [39,41]	$3.70\times 10^{-20}$ ^a^
$\zeta_{i}$, mV	−15.0 [52]	−35.6 [53]	−17.1 [12]	−44.2 [53]	+ 45 [54]	0 ^b^

^a^ assumed to be identical to pure water [41]. ^b^ Pure water is not conductive ($\zeta_{i}=0\;\mathrm{mV}$).

## Data Availability

Data is contained within the article.
